# ADA as main biochemical marker in patients with tuberculous effusion

**DOI:** 10.5937/jomb0-44018

**Published:** 2023-10-27

**Authors:** Jelena Janković, Branislav Ilić, Nataša Đurđević, Aleksandar Jandrić

**Affiliations:** 1 University Clinical Center of Serbia, Clinic for Pulmonology, Belgrade; 2 University of Belgrade, Medical Faculty, Belgrade

**Keywords:** adenosine deaminase, biomarkers, pleural effusion, tuberculosis, adenozin deaminaza, biomarkeri, pleuralni izliv, tuberkuloza

## Abstract

Tuberculous pleuritis (TP) is one of the most common extra-pulmonary tuberculosis form. Because of tuberculous pleurisy is hard to diagnose due to slow course of disease and lack of specificity in symptoms and diagnostic methods. In that reason, we need multidisciplinary approach and efficient biomarkers. Acid-fast bacilli (AFB) staining, cultures and pathophysiological biopsy finding from the majority of patients are positive only in less than 10%. Löwenstein culture results need time about 6-8 weeks what delays diagnosis. Adenosine deaminase (ADA) is biomarker with high sensitivity and specificity (more than 90%) and considered as gold standard of biomarkers in the diagnosis of TP. It is very hard to distinguish malignant from TP with lymphocyte predomination, but in patient with malignant pleural effusion the level of ADA is decreased, opposite from TP. ADA in pleural punctate is a fast, simple, efficient and economical way for clarification the etiology of the pleural effusion as tuberculous pleurisy. Also, many studies have proved the role of ADA in the response to treatment for tuberculosis at follow up period.

## Abstract (sr)

Tuberkulozni pleuritis (TP) je jedan od najčešćih oblika vanplućne tuberkuloze. Zbog sporog toka bolesti i nespecifičnosti simptoma i dijagnostičkih metoda teško je dijagnostikovati tuberkuloznu pleuritis. Iz tog razloga NAM je potreban multidisciplinarni pristup i efikasni biomarkeri. Acidoalkoholno rezistentan bacil (AFB), kulture i nalaz patofiziološke biopsije kod većine pacijenata su pozitivni samo kod manje od 10%. Za rezultate Lovenstein kulture potrebno je vreme oko 6-8 nedelja, što odlaže dijagnozu. Adenozin deaminaza (ADA) je biomarker visoke osetljivosti i specifičnosti (vi{e od 90%) i smatra se zlatnim standardom biomarkera u dijagnozi TP. Veoma je teško razlikovati maligni od TP sa dominacijom limfocita, ali kod pacijenata sa malignim pleuralnim izlivom nivo ADA je smanjen, za razliku od TP. ADA u pleuralnom punktatu je brz, jednostavan, efikasan i ekonomičan način za pojašnjenje etiologije pleuralnog izliva kao tuberkuloznog pleuritisa. Takođe, mnoge studije su dokazale ulogu ADA u odgovoru na lečenje tuberkuloze u periodu praćenja.

## Introduction

Pleural fluid is an ultra-filtrate of blood serum that is produced on the parietal and absorbed on the visceral pleura [1]. Normally, there is a physiological amount of fluid in the pleural cavum to prevent friction during the breathing process. Production and absorption (which can be up to 20 times higher capacity) must be in balance. If the fluid accumulation exceeds physiological values (0.2 mL/kg/h), a pleural effusion (PE) occurs [1]. Pathogenesis of pleural PE are: low oncotic pressure or elevated hydrostatic pressure of the systemic circulation, due to elevated pulmonary capillary pressure, increased permeability or lymphatic obstruction [1]. The causes can be pulmonary and extra-pulmonary, and depending on the location, they can be unilateral or bilateral. Symptoms depends on the amount and speed of effusion formation [2]. The disease can be asymptomatic when it is most often detected as part of the diagnosis of other diseases, or manifest with symptoms of cough, dyspnea, chest pain or hemoptysis [2]. More than 100 diseases can be manifested by the image of pleural effusion. It is more common in pneumonia, pulmonary embolism, heart failure, kidney failure, as part of malignancy (lung, ovary, breast, pancreas), in Dresler’s syndrome and others. A multidisciplinary approach is required for diagnosis, differentiation of the cause of effusion and treatment. The first step in diagnosis is radiological – chest X-ray, ultrasound and computed tomography. The sample for analysis was obtained by thoracentesis. Pleural effusion is analyzed microbiologically (bacteriologically, for viruses, cultivation), serologically, cytology and biochemically [2] [3]. The use of different biomarkers can help to quickly and efficiently clarify the etiology of the effusion and decide on treatment. Elevated values of CRP and procalcitonin indicate para-pneumonic pleural effusion; for heart failure elevation of pro-BNP values, amylase for pancreatitis, immunological spectrum of analyzes for systemic diseases [1]. Biomarkers for malignant effusion are classified into: those based on soluble, immuno-cytochemical and biomarkers based on nucleic acids. The punctate is suitable for the analysis of EGFR mutations and the effectiveness of TKI in patients with non-small cell carcinoma when tumor biopsy is not possible [4].

There is a big problem in differentiation tuberculous from other pleural effusions. The aim of this review paper is to clarify the diagnosis of TP and the importance of an adequate simple and useful biomarker due to the lack of other diagnostic procedures.

### Tuberculous Pleuritis

Tuberculosis (TB) is the leading cause of death, from all infectious diseases, with approximately 1.4 million people in 2019. The extra-pulmonary form takes a 25% of patients with TB. Lymphadenitis and tuberculous pleuritis (TP) are the commonest extra-pulmonary TB forms. By literature, two-thirds of people with TP will progress to a pulmonary form of tuberculosis within 2 years if they were not treated properly. A small percent of them will develop severe complications, such as empyema, bronchopleural fistulas or fibrothorax [5].

Tuberculous pleuritis occurs when Mycobacterium tuberculosis antigen release from ruptured a subpleural caseous focus go into the pleural space. The initial inflammatory reaction lids to capillary permeability, influx of proteins and higher rate of pleural fluid formation [6]. The Mycobacterial antigen in the pleural fluid elicits an intensive inflammatory response with cellular components in. TP characterized with rapid influx of neutrophils, followed by macrophages and after with lymphocyte population mainly T-helper type 1 (Th1). That is lymphocyte-predominant exudative pleural effusion [6] [7]. The problem is when a thoracentesis is obtained at an early stage when there is a predominance of neutrophils. Then there may be a suspicion of a parapneumonic effusion and correlation with the clinical picture and other biomarkers such as C-reactive protein are required [8]. Also, problem can be with malignant pleural effusion which also is lymphocyte predominance. We need a biomarker which can differentiate malignant form tuberculous pleural effusion, or different TE from others.

### Diagnosis of TP

Tuberculous pleurisy is hard to diagnose due to slow course of disease and lack of specificity in symptoms and diagnostic methods. Because of that is need multidisciplinary approach [9]. Symptoms are usually acute or subacute: 75% of patients have chest pain, 70% cough, 85% night sweats, 50% dyspnea and more than half with fever [9]. So, symptoms are not typical and need other diagnostic tools. Many of those patients doesn’t have lung changes at first, so sputum for Ziehl–Neelsen staining can be negative. Better approach is thoracentesis. Tuberculous pleural effusion is an exudate, colorless or straw (yellowish) or sometimes can also be bloody [9]. Neutrophils are dominant in the initial 24 h of the inflammatory response, macrophages are in peak at 96 h after the onset of inflammation, and T lymphocytes are dominant in the subsequent inflammatory response, gradually forming pleural granulomas [9].

Diagnosis of TP met the following criteria: detection acid-fast bacilli (AFB) staining or Löwenstein–Jensen pleural fluid cultures, pleural biopsy culture and histology (granuloma-like changes in pleural biopsy samples and exclusion of pleurisy from other causes [10] [11].

Most of these analysis have a one or more limitations. Acid-fast bacilli staining and cultures from the majority of patients are negative, only less than 10% of those are positive for AFB [10] [12]. For Löwenstein–Jensen culture results need time about 6-8 weeks what delays diagnosis and starting treatment. And results are less than quarter positive [13]. Several studies from literature, have evaluated Xpert MTB/RIF assay using pleural fluid and high cost of the tests. These studies showed sensitivity ranging from 15% to 44% [10]. Limitation for thoracentesis is small sample for diagnosis (few syringes) if we know that in pleural cavity is much more effusion, so the sample cannot be typical representative for entire amount and characteristics of pleural fluid.

Pleural biopsy approach is invasive and usually negative because it is blind closed biopsy, also cannot be performed in all hospitals [14]. Cytology for differentiation TP from malignant is hard, both are with lymphocyte predomination. We need cytology for malignant cells but sensitivity for pleural malignancy is only average 62% [10]. More invasive approach is pleuroscopy in sedation or VATS with many contraindications and complications (pneumothorax). Sensitivity and specificity are 91% and 100% [13].

Because of all of the above, the road to diagnosis TP is difficult and it is wasted a lot of time to a right diagnosis and start for treatment. A biomarker with high sensitivity and specificity is needed, with a practically non-invasive approach and excellent results.

### Ada as a main biomarker of TP

The biomarker of choice, for diagnosis TP, is ADA (adenosine deaminase). This biomarker has high specificity and sensitivity (over 90%) and is considered the gold standard of biomarkers in the diagnosis of TP [10]. In 2019, Aggarwal et al. [15] updated the sensitivity (0.92) and specificity (0.90) of ADA with conclusion as good biomarker for detecting pleural TB among adults, including 174 publications. Systematic review and meta-analysis also showed that ADA is good for detecting pediatric TP [16].

ADA is an enzyme synthesized by many cells such as mononuclear cells, lymphocytes, neutrophils and is often associated with intracellular infections such as tuberculosis. On basis of the elevated level of ADA in the pleural effusion, TP can most often be distinguish from parapneumonic pleurisy, although high ADA values have been recorded in empyema. In those cases, the type of cells in the pleural effusion plays a key role, where neutrophils dominate in empyema, and lymphocytes in TP. In patients with malignant pleural effusion, the level of ADA is decreased, although the diagnosis of malignant pleural effusion cannot be made based on the level of ADA alone [17].

There are two different types of ADA biomarker: ADA1 and ADA2. ADA1 is ubiquitous and can be found in many cells, but ADA2 is produced by monocyte/ macrophages and is responsible for tuberculous pleuritic [7]. Mycobacterial antigens stimulate T lymphocytes in pleural fluid. ADA is a T lymphocyte enzyme that catalyzes adenosine into inosine and because of that the amount of this enzyme is increased in TP as a lymphocyte-rich exudate [14]. High-ADA levels are in TP but malignant pleural effusions usually have low ADA levels [7] [18].

The most accepted cutoff value for pleural ADA is 40 U/L (10). One-third of para-pneumonic effusions and 70% of empyemas have ADA levels above 40 U/L, they can be distinguished from TP by the clinical presentation and because they are neutrophils predominant fluids. High pleural fluid ADA levels have also been reported in more than half of effusions in patients with lymphomas, but this effusion have extremely high ADA values (>250 U/L). [7]. High level of ADA in pleural fluid has been reported in malignancies, pneumonia, infectious mononucleosis, rheumatoid arthritis or systemic lupus erythematosus, granulomatous inflammation, pericardial effusion, which causes frequent false-positive results. Because of that, by literature and meta-analysis, ADA2 as isozymes may help distinguish TP from other types of pleural effusion [10] [19].

Many studies, as well as meta-analysis, showed that ADA2 has better diagnostic accuracy and greater sensitivity and specificity in the diagnosis of TP, but further research is needed to show the place of importance of routine ADA2 determination in clinical practice [19].

The level of ADA in the pleural effusion can be lowered in the elderly, the critical ill, as well as in patients with multiorgan dysfunction. Therefore, a low level of ADA does not completely rule out TP, and it is necessary to be very careful in interpreting the level of ADA in the mentioned situations, especially if there is a clinical suspicion of tuberculosis [20].

Numerous studies have been conducted on different populations, such as Spanish, Chinese, Brazilian, but the results agree that the sensitivity and specificity for TP are over 90% [21].

In high TB prevalence regions and patients with presence of a lymphocyte-predominant exudate with clinical suspicion of TB and ADA value of >40 IU/L has a positive predictive value of 98%. Otherwise, in low prevalence areas, the normal or low ADA values and lymphocyte predominance makes TB very unlikely. Than pleural biopsy need to be performed to confirm the diagnosis of TP [19].

Although numerous studies show the diagnostic importance of ADA in the diagnosis of different types of pleural effusions, there are still many controversies. First, around the pleural ADA cutoff value. As already mentioned, the most common cutoff for pleural ADA in the diagnosis of TP is 40 U/L. However, it was shown that the cutoff depends on the prevalence of tuberculosis in the investigated region. In regions with high prevalence, pleural ADA values above 20 U/L showed excellent sensitivity and specificity, while in regions with low or decreasing prevalence, pleural ADA values between 40 U/L and 70 U/L may be associated with numerous false positives. Results, and the authors suggest a cut off of 70 U/L [22]. Because of that, the level of pleural ADA is increasingly used as part of various ratio or scoring systems.

One of the most commonly used ratios is the serum LDH/pleural ADA (cancer ratio), where values over 20 suggest a malignant pleural effusion. Also, the pleural LDH/pleural ADA ratio has great diagnostic significance in diagnosing TP and distinguishing TP from parapneumonic pleural effusion. Another study found that the LDH/ADA ratio in pleural fluid was highly predictive of distinguishing TPE from PPE at a cutoff level of 16.2 [23].

In addition to the LDH/ADA ratio, the level of ADA in the pleural effusion is also part of the scoring system in the diagnosis of pleural effusions, which also includes pleural LDH, LDH/ADA ratio, the level of serum albumin and albumin in the pleural punctate, the cell type of the pleural punctate, etc. In a Chinese retrospective study, it was shown that cut-off values of ADA > 19.65 U/L, LDH/ADA 29.61 and S-Alb > 23.95 g/L show 100% sensitivity and 98.7% specificity for differential diagnosis TP [23].

Many studies have proved the role of ADA in the response to treatment at follow up period. The disadvantage of this biomarker is that it does not provide information on cultivation, i.e. type of mycobacteriosis and drug sensitivity and resistance. In a small prospective study from India, the authors showed that the level of serum ADA can be useful for monitoring the therapeutic effect of antituberculosis treatment. It was proved that there was a significant difference between serum ADA levels before and after the intensive phase of tuberculosis treatment (P < 0.001). Further studies are needed to eventually confirm these findings and assess whether the serum ADA level test can be used to assess the response to antituberculosis treatment in daily clinical practice [24] [25].

## Conclusion

ADA is very sensitive and specific biomarker, it is available and useful, so, ADA should be used whenever possible. ADA in pleural punctate is a fast, simple, efficient and economical way for clarification the etiology of the pleural effusion as tuberculous pleurisy. In this way, faster diagnostics will be enabled without the application of more invasive diagnostic methods, as well as targeted and effective treatment. Although ADA is not the gold standard test for the diagnosis of TPE, it is recommended as a ‘rule out’ test in countries with a low prevalence of TB and »rule- in« test in countries with a high prevalence of TB [21].

## Dodatak

### Acknowledgements

All authors have contributed equally towards the conception of the manuscript.

### Authors’ Contributions

All authors contributed to the study conception and design. Data collection and analysis were performed by Jelena: Jankovic, Branislav Ilic and Natasa Djurdjevic. The first draft of the manuscript was written by Jelena Jankovic and all authors commented on previous versions of the manuscript. For radiological finding contributed Aleksandar Jandric. Critical revision of manuscript: Natasa Djurdjevic and Aleksandar Jandric. All authors read and approved the final manuscript.

### Conflict of interest statement

All the authors declare that they have no conflict of interest in this work.
